# Implementation of an evidence-based management algorithm for patients with chronic pancreatitis (COMBO trial): study protocol for a stepped-wedge cluster-randomized controlled trial

**DOI:** 10.1186/s13063-022-07044-8

**Published:** 2023-01-07

**Authors:** Florence E. M. de Rijk, Charlotte L. van Veldhuisen, Marc G. Besselink, Jeanin E. van Hooft, Hjalmar C. van Santvoort, Erwin J. M. van Geenen, Cornelis H. van Werkhoven, Pieter Jan F. de Jonge, Marco J. Bruno, Robert C. Verdonk, for the Dutch Pancreatitis Study Group

**Affiliations:** 1 Department of Gastroenterology and Hepatology, Erasmus University Medical Center, Doctor Molewaterplein 40, 3015 GD Rotterdam, The Netherlands; 2 Department of Research and Development, St. Antonius Hospital, Nieuwegein, The Netherlands; 3 Department of Surgery, Amsterdam UMC, Location University of Amsterdam, Amsterdam, The Netherlands; 4 Amsterdam Gastroenterology Endocrinology Metabolism, Amsterdam, The Netherlands; 5 Department of Gastroenterology and Hepatology, Leiden University Medical Center, Leiden, The Netherlands; 6 Department of Surgery, St. Antonius Hospital, Nieuwegein, The Netherlands; 7 Department of Surgery, University Medical Center Utrecht, Utrecht, The Netherlands; 8 Department of Gastroenterology and Hepatology, Radboud University Medical Center, Nijmegen, The Netherlands; 9 Julius Center for Health Sciences and Primary Care, Utrecht, The Netherlands; 10 Department of Gastroenterology and Hepatology, St. Antonius Hospital, Nieuwegein, The Netherlands

**Keywords:** Chronic pancreatitis, Management algorithm, Integrated care, Evidence-based, Quality of life, Pain severity, Stepped-wedge, Randomized controlled trial, COMBO

## Abstract

**Background:**

Chronic pancreatitis (CP) is an inflammatory disease that may be complicated by abdominal pain, pancreatic dysfunction, nutritional deficiencies, and diminished bone density. Importantly, it is also associated with a substantially impaired quality of life and reduced life expectancy. This may partly be explained by suboptimal treatment, in particular the long-term management of this chronic condition, despite several national and international guidelines. Standardization of care through a structured implementation of guideline recommendations may improve the level of care and lower the complication rate of these patients. Therefore, the aim of the present study is to evaluate to what extent patient education and standardization of care, through the implementation of an evidence-based integrated management algorithm, improve quality of life and reduce pain severity in patients with CP**.**

**Methods:**

The COMBO trial is a nationwide stepped-wedge cluster-randomized controlled trial. In a stepwise manner, 26 centers, clustered in 6 health regions, cross-over from current practice to care according to an evidence-based integrated management algorithm. During the current practice phase, study participants are recruited and followed longitudinally through questionnaires. Individual patients contribute data to both study periods. Co-primary study endpoints consist of quality of life (assessed by the PANQOLI score) and level of pain (assessed by the Izbicki questionnaire). Secondary outcomes include process measure outcomes, clinical outcomes (e.g., pancreatic function, nutritional status, bone health, interventions, medication use), utilization of healthcare resources, (in) direct costs, and the level of social participation. Standard follow-up is 35 months from the start of the trial.

**Discussion:**

This is the first stepped-wedge cluster-randomized controlled trial to investigate whether an evidence-based integrated therapeutic approach improves quality of life and pain severity in patients with CP as compared with current practice.

**Trial registration:**

ISRCTN, ISRCTN13042622. Registered on 5 September 2020.

**Supplementary Information:**

The online version contains supplementary material available at 10.1186/s13063-022-07044-8.

## Introduction

Chronic pancreatitis (CP) is a multifactorial inflammatory disease in which recurrent episodes of inflammation lead to irreversible pancreatic tissue damage and consequently loss of function. CP is often complicated by abdominal pain, exocrine/endocrine pancreatic insufficiency, and anatomic complications (e.g., duodenal or biliary stenosis, splanchnic venous thrombosis, pseudocysts, and pseudoaneurysmata) which negatively impact patients’ quality of life (QoL) and life expectancy [1–8].

Recommendations regarding the diagnosis and therapy of CP (and its complications) are summarized in the evidence-based European HaPanEU guidelines [9]. However, current adherence to these guidelines is suboptimal, which leads to undertreatment and practice variety [10, 11]. This may partly explain the impaired QoL frequently observed in patients with CP. Thus, improvements are clearly needed to improve patients’ outcomes.

In the past decades, several interventions have been studied in CP (e.g., PERT, nutritional supplements, different endoscopic and surgical strategies for painful CP, bone health management, and consultation). To date, each of these interventions separately, let alone as an integrated management algorithm, is poorly implemented in current daily practice [11]. Optimal care, however, is never a solitary intervention, but usually includes a bundle of measures. At the same time, a bundle of interventions is not necessarily associated with improved outcomes despite evidence for the efficacy of its individual components. The COMBO trial aims to assess to what extent patient education and a structured implementation of an evidence-based algorithm including multiple interventions improve clinical outcomes in patients with CP. We hypothesize that a bundled intervention results in lower pain scores and complication rate, ultimately leading to an improved QoL, social participation, and reduction of health care costs.

## Methods

**Fig. 1:**
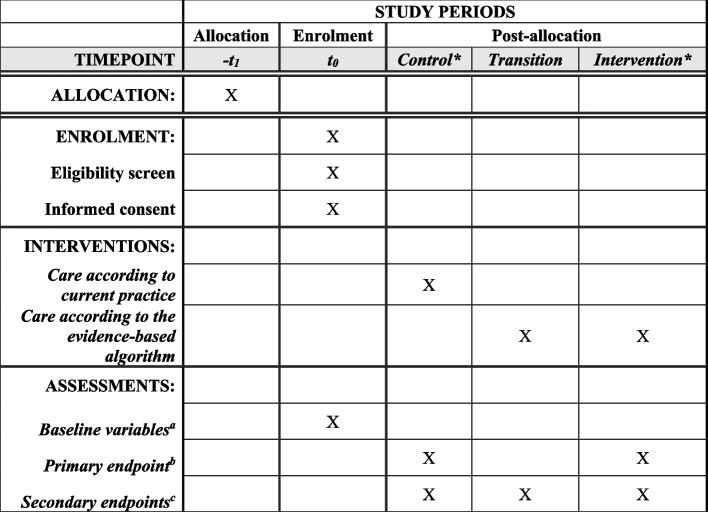
Spirit flow diagram of enrolment, interventions, and assessments for the COMBO study, a stepped-wedge cluster-randomized controlled trial. *Number of outpatient clinic visits varies between patients and depends on the stepped-wedge design of this trial. ^a^Baseline variables: age, sex, center, nicotine and alcohol use, co-morbidity, etiology of chronic pancreatitis, disease duration, prior endoscopic- and/or surgical interventions, pancreatic function and medication use. ^b^Co-primary endpoints: mean difference in quality of life (PANQOLI) and level of pain (Izbicki Pain Score) at 12 months after the end of the transition period for each cluster (intention-to-treat analysis) and at 12 months after start intervention for the individual patient defined as the first outpatient clinic visit after the kick-off meeting has been performed (per-protocol analysis). ^c^Secondary endpoints comprise the following domains: (1) individual components of the co-primary endpoints, (2) process measure outcomes, (3) clinical outcomes (e.g., pancreatic function, nutritional status, bone health), (4) utilization of healthcare resources, and (5) social participation and are collected from patient records at 12 months after the end of the transition period for each cluster (intention-to-treat analysis) and at 12 months after start intervention for the individual patient defined as the first outpatient clinic visit after the kick-off meeting has been performed (per-protocol analysis)

The trial protocol is written in accordance with the CONSORT guidelines for cluster-randomized trial and draft extension for stepped-wedge trials and with the Standard Protocol Items: Recommendation for Interventional Trials (SPIRIT) guidelines (see Fig. 1 and the Additional file 1: Supplementary Appendix 1: SPIRIT checklist) [12, 13].

### Study design and setting

**Fig. 2:**
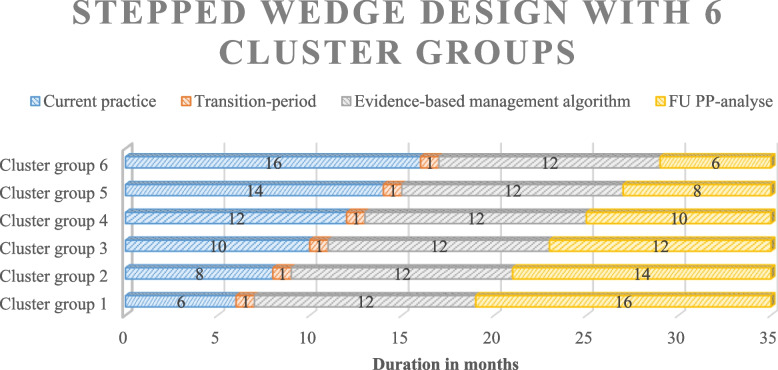
Stepped-wedge design with 6 cluster groups. No cluster groups receive the intervention at baseline (6 months); all cluster groups start with continuing current practice. Cluster groups are randomly assigned to unidirectional cross-over to receive the intervention at 2-month intervals. Total trial duration will be a maximum of 35 months; a baseline period of 6 months, a current practice period of varying in length, a transition period of 1 month, an intervention period of 12 months, and a follow-up period with a minimum duration of 6 months for those patients participating in the per-protocol analysis

The COMBO trial is a nationwide stepped-wedge cluster-randomized controlled superiority trial enrolling CP patients who are receiving active treatment in one of the participating hospitals at time of inclusion. In total, 26 centers of the Dutch Pancreatitis Study Group (DPSG) are participating in this trail. In a stepwise manner, an evidence-based algorithm for the treatment of CP is implemented in all participating centers. Participating centers are divided into 6 clusters, based on location and referral pattern for patients with CP according to the current situation in the Netherlands. Each cluster contains 4 or 5 DPSG centers, among which at least one academic or non-academic teaching hospital and its regional affiliated (non-)teaching hospitals. At the start of the trial, all clusters continue current practice (i.e., control) for at least 6 months, which is considered as the baseline period. Thereafter, clusters are randomly assigned to unidirectional cross-over to the intervention period (i.e., care according to the evidence-based algorithm) at 2-month intervals. Following this fashion, the trial proceeds until all clusters have crossed over to the intervention phase and delivered the intervention for at least 18 months. Total duration of this trial is 35 months (see Fig. 2).

### Eligibility criteria

Study participants are recruited from 26 centers of the DPSG during the current practice phase of each cluster.

#### Inclusion criteria

The inclusion criteria are as follows:

- Diagnosis of CP according to the M-ANNHEIM criteria [14]
- Age ≥ 18 years
- Active treatment in one of the participating hospitals (i.e., annual checkup appointment for CP)
- Provided written informed consent (IC)

#### Exclusion criteria

The exclusion criteria are as follows:

- Pregnancy
- End-stage diseases (< 6 months estimated survival) due to cancer, chronic obstructive pulmonary disease, and/or congestive heart failure
- Suspected or established pancreatic malignancies
- Uncompensated liver cirrhosis
- Renal failure (GFR < 25 ml/min or patients receiving dialysis treatment)

### Withdrawal and replacement of participants and centers

Subjects can leave the study at any time for any reason should they wish without any consequences. In order to prevent an unequal distribution of centers across the two study arms (pre-implementation arm versus post-implementation arm), it is essential that all participating centers complete the full study. There will be no replacement of individual subjects and centers after withdrawal.

### Intervention: integrated evidence-based algorithm for the treatment of CP

#### Construction

The evidence-based algorithm is based on the United European Gastroenterology evidence-based guidelines for the diagnosis and therapy of CP (HaPanEU, 2017) and covers the most relevant aspects with respect to prevention and treatment of disease progression and its complications [9]. Guideline recommendations graded as “strong” and/or of high-quality evidence (GRADE) are included in this algorithm [15]. Furthermore, an extensive literature search is performed to identify studies reporting on new interventions for CP that were published since composition of these guidelines (see Additional file 1: Supplementary Appendix 2: systematic literature search). The intervention components included in the algorithm are related to four main domains of disease management: (1) lifestyle modifications and psychological support, (2) pancreatic function, (3) nutritional support, and (4) pain management. Education of both patients and physicians is an important aspect of this algorithm. Therefore, educational brochures (e.g., influence of smoking and alcohol on disease progression, dietary advice) and educational videos (e.g., exocrine pancreatic insufficiency (EPI) and PERT, bone health, endoscopic and surgical treatment of CP) are distributed among patients during this trial. At each participating center, a kick-off meeting is hosted before the start of the intervention phase (i.e., transition period) to inform physicians about the content of the evidence-based algorithm. For each domain, flowcharts are constructed and handed out to all local treating physicians during the intervention phase of this trial. The content of these treatment flowcharts has been critically reviewed by both national and international experts in the field of CP, before incorporating in the study protocol. By applying these flowcharts in clinical practice an individualized and standardized treatment plan will follow. In order to optimize the clinical use of these flowcharts, a web application is especially designed for this trial: https://flowchart.combo-studie.nl (only available in Dutch). This website is freely accessible to physicians after their cluster has crossed over to the intervention phase. No patient-sensitive data traffic runs through this website. The web application is designed to serve as a supportive tool for physicians to provide care according to the evidence-based algorithm, and also allows us to monitor adherence to our treatment algorithm. However, the treating physician is responsible for the application, interpretation, and treatment if indicated. Outpatient follow-up visits take place at the discretion of the responsible physician. A detailed description of the content of the evidence-based algorithm is provided in Additional file 1: Supplementary Appendix 3. The most important recommendations related to the four main domains are listed below. Before the start of this trial, all participating hospitals have indicated their intention to implement the evidence-based algorithm when the randomization order dictates without being aware of the details of this algorithm.

#### Lifestyle modifications and psychological support

Tobacco and alcohol are both identified as independent risk factors for accelerating disease progression in CP [16]. Therefore, appropriate counselling needs to be offered to patients with persistent alcohol and smoking behavior. This includes clinician recommendations to quit smoking and drinking completely, and to hand out patient information leaflets, especially focusing on patients with CP. These leaflets comprise information on the effects of alcohol and tobacco on disease progression, and patients’ outcomes and a self-help manual to quit smoking and drinking. Furthermore, physicians are instructed to advise current alcohol and tobacco consumers to seek for supportive treatment in primary care.

#### Pancreatic function

Exocrine pancreatic function

Diagnostic work-up for EPI requires a non-invasive pancreatic function test (PFT), together with an evaluation, including clinical symptoms (i.e., maldigestion related symptoms) and nutritional status (laboratory blood test). If two out of these three are suspected for EPI due to the presence of symptoms or deviating values, EPI is very likely to be present [4]. In this trial, patients with CP are screened annually for the presence of EPI. Apart from this, a function test is advised in case of new or worsening symptoms of PEI, even when this function test was previously normal [9]. In daily practice, the fecal elastase-1 (FE-1) test is the most commonly performed non-invasive PFT (cut-off used in this trial < 200 µg/g). In case of EPI, enzyme supplementation (PERT) is indicated. As part of the COMBO trial, all PERT users are offered to participate in a 2-year PERT-homecare program (on a voluntary basis), including home visitations and telephone consultations by trained nurses, individual dietary advice, advice on administration of PERT, and supplementary material with extra information regarding EPI and PERT. The aim of this program is to improve patient compliance with PERT and to establish a more individually tailored supplementation plan (i.e., dose adjustments based on the amount of fat intake) in order to optimize PERT.

Endocrine pancreatic function

Initial evaluation for the presence of diabetes mellitus includes fasting plasma glucose (FPG) (≥ 7.0 mmol/l) and HbA1c (> 48 mmol/mol). As proposed by our national diabetes mellitus guideline, an abnormal FPG or HbA1c requires further evaluation by repeat testing or an oral glucose tolerance test (Gold Standard). In this trial, CP patients, not (yet) diagnosed with diabetes mellitus, are screened at least once a year for new-onset diabetes mellitus. In case of new-onset diabetes, additional laboratory tests (e.g., type I associated antibodies (GAD, IA2) and C-peptide/glucose ratio) are indicated to determine the accurate type of diabetes mellitus (diabetes mellitus type I/II or pancreatogenic diabetes) and initiate proper treatment [17]. Type I and type II diabetes are treated according to the current available clinical practice guidelines. Pancreatogenic diabetes is lacking international treatment guidelines. Therefore, this type of diabetes is treated according to the discretion of the local physicians.

#### Nutritional support and bone health

Nutritional status

In the present study, nutritional status is evaluated at each follow-up visit by using the malnutrition universal screening tool (MUST). Furthermore, patients are screened at least once a year for deficiencies of fat-soluble vitamins (vitamin A, D, E and K), minerals (magnesium, zinc, calcium, selenium and iron), and albumin. Patients at high risk of malnutrition are referred to a dietician for dietary consultation and additional support as proposed by the HaPanEU guidelines [9].

Bone health

Patients with CP are at risk for osteoporosis and osteopenia. Therefore, regular screening is recommended [3]. In this trial, a dual-energy X-ray absorptiometry (DXA) is advised in patients with (1) no DXA scan history during the past 5 years or (2) osteopenia and a DXA scan performed more than 2 years ago. In case of osteopathy, patients are treated accordingly as proposed by current guidelines.

#### Pain management

The initial management of pain in patients with CP consists of lifestyle modifications and optimal medical management. In some patients, however, this conservative approach fails and more invasive treatment may be offered, such as patients with long-term need of opioids or specific morphologic abnormalities (e.g., pancreatic ductal stones, strictures, and/or symptomatic pseudocysts). In the present study (i.e., intervention phase), these patients are discussed by a multidisciplinary team. If such local multidisciplinary team meetings do not exist, patients are referred to the Dutch Chronic Pancreatitis Expert Panel for consultation. The Dutch Chronic Pancreatitis Expert Panel includes surgeons, gastroenterologists, pain specialists, and radiologists, all specialized in the field of CP. This pre-existing panel was already in use within the ESCAPE trial where its support proved to be of value [18]. Consultation of this Expert Panel is possible 24 h/day, 7 days a week [19, 20]. The goal of multidisciplinary discussion is to identify those patients who may benefit from an (early) endoscopic or surgical intervention.

#### Implementation

The coordinating investigators will implement the algorithm in each center with the intention of training all local treating physicians on the rationale and use of the algorithm. To ensure adequate implementation of this algorithm, kick-off visits will be performed in all clusters by at least one coordinating investigator during the transition period of their cluster group. In addition, an online-newsletter will be sent every month containing a progress report, link, and reminder for the web application tool and educational videos.

### Primary endpoints

The co-primary outcomes of this study are QoL assessed with the Pancreatitis Quality of Life Instrument (PANQOLI) questionnaire and pain severity assessed according to the Izbicki pain score. PANQOLI is specifically developed and validated for the evaluation of QoL in CP and consists of four subscales: emotional function, role function, physical function, and “self-worth” [21]. This last subscale is unique, which differentiates PANQOLI from the previously applied questionnaires like the SF-12/SF36 and QLQ-C30/QLC-PAN26. Since there is no high-quality data on the dynamics of this score yet, QoL is also assessed by SF-36 in order to compare our results to previously performed studies. The Izbicki pain score consists of four questions regarding frequency of pain, intensity of pain (VAS-score), use of analgesics, and disease-related inability to work. Since there is a large spread in both QoL and pain severity scores among patients with different consequences for each patient’s perspective, both primary study endpoints will be evaluated separately as co-primary endpoints and not as a composite of both.

### Secondary endpoints

Secondary endpoints are provided in Additional file 1: Supplementary Appendix 4 and comprise the following domains: (1) individual components of the co-primary endpoints, (2) process measure outcomes, (3) clinical outcomes (e.g., pancreatic function, nutritional status, bone health), (4) utilization of healthcare resources, and (5) social participation.

### Sample size calculation

Sample size calculation is performed for the primary analysis population and is based on detecting the minimal clinically relevant difference (target difference) in the average PANQOLI scores and the Izbicki pain scores between the two study groups (current practice versus intervention). The protocol committee agreed by consensus on a clinically relevant difference of 10 points on the Izbicki pain score on average. Based on previously performed studies, we assumed a mean difference of 10% in QoL scores [22–24].

No high-quality data on the dynamics of PANQOLI scores have been reported yet; therefore, the Physical Component Summery scores of the SF36 (SF36_PCS) are used for simulations as being the best proxy. The intervention is considered successful if either or both pain and QoL scores improve. Therefore, to adjust for multiple testing, a two-sided alpha of 0.025 is used. We used data from the CARE registry to calculate our sample size [19]. The Izbicki pain score required a larger sample size as compared to SF36_PCS. This is mainly due to a larger within-patient variability of both intercept and slope. The model and parameters we used to determine the intra-patient variability and random error for the Izbicki pain score and to calculate the sample size are provided in Additional file 1: Supplementary Appendix 5. Based on this design, with 120 subjects the power to demonstrate an average slope change of 10 points reduction after 365 days is > 90%.

### Randomization and blinding

Clusters are randomized using R statistical software to determine the timing of cross-over. Participating centers are recruited before randomization. Randomization is performed during the baseline period at a single point by the independent trial statistician who is not involved in the conduct of the trial. The cross-over date is revealed sequentially to the centers (i.e., local principle investigators) as they approach the time of cross-over. The local principal investigators are blinded to the randomization sequence for all other participating centers.

### Follow-up

The follow-up duration is 35 months from the start of this trial. Data regarding the co-primary endpoints are collected through questionnaires at 3-month intervals from the start of this trial until the end of follow-up. Furthermore, every patient receives an extensive questionnaire for a total of four measurement points (at 3 months before start intervention, at start intervention, at 6 months after start intervention and at 12 months after start intervention) to collect information on secondary endpoints (i.e., symptoms of CP, work ability, healthcare utilization, current alcohol and smoking behavior). Data collected from patients’ surveys are checked with source data from patient records.

### Statistical analysis

To evaluate the effect of this algorithm in terms of improving QoL and reducing pain severity, the scores of patients before implementation of our algorithm (i.e., current practice) will be compared to those of patients obtained after implementation of the integrated management algorithm. The effect of the intervention will be measured in all participants according to the intention-to-treat principle, meaning that patients will be analyzed as exposed to the intervention according to the order and dates of the randomization schedule. Secondary analyses include a per-protocol analysis in which the outcomes of patients actually receiving the intervention (defined as an outpatient clinic visit within ≤ 6 months after the cluster has switched over to the intervention) will be evaluated. The effect of the intervention will be expressed as a step-change (i.e., immediate effect of the intervention) and slope-change (i.e., effect of the intervention on the secular trend). Time is a potential confounder and changes external to the trial may create underlying secular trends. Therefore, analyses will be adjusted for time effects irrespective of their statistical significance.

#### Primary and secondary analysis populations

In this study, all CP patients who meet the inclusion criteria, regardless of their time since diagnosis, are eligible for participation. However, the primary analysis study population consists of patients with a time since diagnosis ≤ 3 years at the start of the intervention. This population is chosen since in these patients the intervention is intended to start early after diagnosis and the investigators postulate that the impact of the intervention may depend on the time since diagnosis. Secondary analysis populations include patients with a time since diagnosis between 4 and 10 years and > 10 years and the total population.

#### Baseline values

Baseline criteria (i.e., at time of inclusion) are age, sex, center, nicotine and alcohol use, co-morbidity, etiology of CP, disease duration, prior endoscopic- and surgical interventions, pancreatic function, and medication use. Baseline values will be analyzed and reported using standard descriptive statistics.

Categorical data will be presented in numbers and percentages. Continuous variables will be summarized as mean with standard deviation (SD) or median with interquartile range (IQR) depending on normality of distribution. Missing baseline data will be handled by using multiple imputation techniques using the fully conditional specification method (aka “chained equations”) assuming data are Missing At Random (MAR). The number of required imputations will be based on the two-stage calculation using a quadratic rule [25]. Missing outcome data will not be imputed; the mixed-effects linear regression analysis is valid despite missing values when assuming MAR.

#### Primary outcomes

The two co-primary outcomes of this study are mean difference in QoL (PANQOLI) and level of pain (Izbicki Pain Score) at 12 months after the end of the transition period for each cluster (intention-to-treat analysis) and at 12 months after start intervention for the individual patient defined as the first outpatient clinic visit after the kick-off meeting has been performed (per-protocol analysis).

For the primary outcomes, analyses will be performed using a mixed-effects linear regression analysis, whereby the intervention is compared to current practice (control). The model includes time since diagnosis in years, intervention (yes/no), and time since intervention in years (which will be zero for observations in the control group). Thus, the intervention effect will be modelled as an interrupted time series analysis with a step-change and a slope-change. When appropriate, patient factors collected at enrolment known to predict QoL and pain, respectively, will be included as co-variates. The estimated effect of the intervention at 12 months after the start of the intervention will be expressed as the sum of the step-change and the slope-change. The confidence interval will be derived using the delta method, i.e., adding up the variances and 2 times the co-variance of the estimated step- and slope-change to derive the variance of the total intervention effect. The step-change and slope-change will be separately reported. The Bonferroni correction method will be used to adjust for multiple testing. For the co-primary endpoint, a two-sided alpha < 0.025 will be considered statistically significant. Effect estimates for the primary endpoints will be presented with 97.5% confidence intervals.

#### Secondary outcomes

Secondary outcomes are collected from patients’ records at 12 months after the end of the transition period for each cluster (intention-to-treat analysis) and at 12 months after start intervention for the individual patient defined as the first outpatient clinic visit after the kick-off meeting has been performed (per-protocol analysis). For the secondary outcomes, data will be analyzed and reported using standard descriptive statistics. Mixed-effects regression analyses will be performed where appropriate. For the secondary endpoints, a two-sided alpha < 0.05 will be considered statistically significant. Effect estimates are presented with 95% confidence intervals.

#### Subgroup and sensitivity analyses

Predefined subgroup analyses will be performed to investigate differences between subgroups for the co-primary and secondary endpoints. Subgroup analyses will be performed based on disease duration, hospital volume, pain severity at baseline, QoL at baseline, and disease etiology. A sensitivity analysis will be performed to evaluate the correlation between success of implementation of the intervention and change in the co-primary endpoint.

#### Economic evaluation

The economic evaluation comprises a cost-effectiveness analysis (CEA) and a cost-utility analysis (CUA). The cost analysis is set up from a healthcare and societal perspective. Three cost categories are analyzed, i.e., healthcare costs (Medical Consumption Questionnaire, MCQ), patient and family costs (Medical Consumption Questionnaire, MCQ), and costs from a societal perspective (Productivity Costs Questionnaire, PCQ). For the cost-utility analysis, costs per additional quality-adjusted life years (QALY) are measured by using the EQ-5D “health utility measure”.

#### Safety and interim analyses

There will be no additional risks for patients participating in this trial. The effectiveness and safety of each intervention separately were previously studied and therefore included in current guidelines. There will be no interim analyses performed for safety and treatment effect. An interim administrative look will be performed to monitor recruitment and retention rate. After 5 months of inclusion, the total number of inclusions will be evaluated. If less than 75% of the sample size is reached at that time, the duration of time between each step will be prolonged for the remaining part of the study to maintain the power of > 90%. More details on strategies for achieving adequate participant enrollment are provided in Additional file 1: Supplementary Appendix 1.

## Discussion

The COMBO trial is the first stepped-wedge cluster-randomized controlled trial designed to determine whether standardization of care through the implementation of an evidence-based algorithm improves clinical outcomes in patients with CP.

Although several interventions have been studied in patients with CP, these were often studied as stand-alone intervention or in selected patients, but not combined as an “integrated management algorithm”. The Dutch multicenter randomized ESCAPE trial compared the effect of early surgery to an endoscopy-first approach on pain in CP patients. In this study, early surgery resulted in significantly less pain over 18 months compared to the step-up approach [20]. Remarkably, this did not lead to an improved QoL. This highlights the need to better understand contributing factors to an impaired QoL in patients with CP [6]. Factors influencing QoL have been extensively investigated in previous studies (e.g., pain pattern and severity, opioid usage, alcoholic etiology, current nicotine and alcohol use, unemployment, EPI, low BMI, bowel symptoms, fear of future, and self-blame) and accounted only for 27 and 18% of the difference in physical and mental health QoL [6, 26–28]. Recently, a mono-center pilot-study has investigated the impact of a dedicated multidisciplinary CP team and has shown promising results in terms of improving QoL and reducing the use of opioids [24]. However, up to now, the impact of an evidence-based algorithm involving multiple domains of disease management on patients’ outcomes has not been evaluated. The COMBO trial will be the first study assessing whether standardization of care through the structured implementation of an evidence-based algorithm improves patient-centered and clinical outcomes in patients with CP and whether it reduces health care and societal costs. This multicenter study is performed on a nationwide level in 26 participating hospitals. The intervention components included in our evidence-based algorithm are based on recommendations stated in society guidelines and therefore we consider them as “state-of-the-art practice” and believe these should be provided to all patients with CP.

By implementing our evidence-based algorithm in clinical practice, we aim to reinforce and improve current clinical care and, subsequently, improve patients’ outcomes. When the intervention (i.e., evidence-based algorithm and its tools) is successfully implemented in all participating centers and provse to be of value in improving QoL and reducing pain severity, it will become the new standard of care for patients with CP.

In the present study, the intervention is implemented in a stepwise manner. Therefore, clusters are randomized instead of individual patients. In the end, the intervention rolls out to all included patients of the participating centers. This study design was chosen for several reasons. First, cluster randomization allows us to measure the impact of our evidence-based algorithm on patients’ outcomes at group level instead of at individual patient level taking into account the complexity of daily care. If individual participants were randomized such as in a traditional randomized controlled trial design, it would be difficult for physicians to avoid contamination, i.e., to not apply in patients randomized to the control group what they learned for the intervention group. Second, we consider our evidence-based algorithm as “best practice” and believe standard CP care should comprise all intervention components of our algorithm. However, for logistical and practical reasons, it is not feasible to introduce the intervention in all participating hospitals at once. By rolling out the intervention in a stepwise manner at cluster level, the intervention will eventually be delivered to all included patients. Furthermore, this stepwise implementation allows to investigate the impact of the intervention over time. For these reasons, we have opted for a stepped-wedge cluster-randomized controlled trial design. One potential drawback of the stepped-wedge design used in this study is the risk of contamination. Introducing this trial in the Netherlands will result in an increased awareness for CP which could possibly lead to higher compliance to the HaPanEU guidelines in the control period. In order to minimize the risk of contamination, the content of the algorithm will only be accessible for local treating physicians in the intervention phase.

In conclusion, the COMBO trial is a stepped-wedge cluster-randomized controlled trial to investigate whether standardization of care by implementing a bundled evidence-based algorithm covering several domains of care improves QoL and pain severity in patients with CP as compared with current practice.

## Trial status

The trial was registered on the 5th of September 2020 in the ISRCTN registry. The first patient was included on the 17th of September 2020. To date, 521 patients have been included in this trial. This trial will run until the 31st of July 2023. The protocol publication has been postponed until all clusters crossed over to the intervention to avoid contamination of the control group.

## Supplementary Information

**Additional file 1: Supplementary Appendix 1. **SPIRIT Checklist. **Supplementary Appendix 2.** Systematic Literature Search. **Supplementary Appendix 3.** Evidence-Based Algorithm. **Supplementary Appendix 4.** Secondary Endpoints. **Supplementary Appendix 5.** Sample size calculation.

## Data Availability

Patients are coded by a numeric randomization code (anonymized), based on the chronological order of inclusion. The source data will be centrally collected and stored at the datacenter of the DPSG (St. Antonius Hospital, Nieuwegein) for a minimum of 15 years. The coordinating investigators (FdR and CvV) are responsible for the implementation and coordination of this trial, patient enrollment, data collection, and data analyses. The results of this trial will be verified by an independent statistician. All coauthors will interpret the data and collaborate on the final manuscript preparation. Study results will be published in a peer-reviewed journal.
