# Supplementary material for: Implementation of an evidence-based management algorithm for patients with chronic pancreatitis (COMBO trial): study protocol for a stepped-wedge cluster-randomized controlled trial
Source: Trials. 2023 Jan 7;24:18. doi: 10.1186/s13063-022-07044-8 (PMC9824955; doi:10.1186/s13063-022-07044-8)
Supplement: Supplementary file 1 — Additional file 1: Supplementary Appendix 1. SPIRIT Checklist. Supplementary Appendix 2. Systematic Literature Search. Supplementary Appendix 3. Evidence-Based Algorithm. Supplementary Appendix 4. Secondary Endpoints. Supplementary Appendix 5. Sample size calculation. [file 13063_2022_7044_MOESM1_ESM.docx]

**SUPPLEMENTARY APPENDICES**

TABLE OF CONTENTS

[SUPPLEMENTARY APPENDIX 1: SPIRIT CHECKLIST 2](#_Toc102050514)

[SUPPLEMENTARY APPENDIX 2: SYSTEMATIC LITERATURE SEARCH 15](#_Toc102050515)

[Life style modifications 15](#_Toc102050516)

[Exocrine pancreatic insufficiency 16](#_Toc102050517)

[Endocrine pancreatic insufficiency 18](#_Toc102050518)

[Nutritional status 20](#_Toc102050519)

[Bone health 21](#_Toc102050520)

[Pain management 23](#_Toc102050521)

[SUPPLEMENTARY APPENDIX 3: EVIDENCE-BASED ALGORITHM 26](#_Toc102050522)

[Exocrine pancreatic function 26](#_Toc102050523)

[Endocrine pancreatic function 28](#_Toc102050524)

[Nutritional status 30](#_Toc102050525)

[Bone health 32](#_Toc102050526)

[Pain management 34](#_Toc102050527)

[SUPPLEMENTARY APPENDIX 4: SECONDARY ENDPOINTS 37](#_Toc102050528)

[SUPPLEMENTARY APPENDIX 5: SAMPLE SIZE CALCULATION 43](#_Toc102050529)

# SUPPLEMENTARY APPENDIX 1: SPIRIT CHECKLIST

SPIRIT 2013 Checklist: Recommended items to address in a clinical trial protocol and related documents*

| Section/item | ItemNo | Description | Addressed on page number |
| --- | --- | --- | --- |
| **Administrative information** | | |  |
| Title | 1 | Descriptive title identifying the study design, population, interventions, and, if applicable, trial acronym | 1 |
| Trial registration | 2a | Trial identifier and registry name. If not yet registered, name of intended registry | 4 |
|  | 2b | All items from the World Health Organization Trial Registration Data Set | 4 (Please see [ISRCTN13042622](https://www.isrctn.com/ISRCTN13042622?q=13042622&filters=&sort=&offset=1&totalResults=1&page=1&pageSize=10) for a more detailed description of the World Health Organization Trial Registration Data Set items) |
| Protocol version | 3 | Date and version identifier | 1 |
| Funding | 4 | Sources and types of financial, material, and other support | 26 |
| Roles and responsibilities | 5a | Names, affiliations, and roles of protocol contributors | 1-2, 24-26 |
|  | 5b | Name and contact information for the trial sponsor | Prof. dr. Marco J. Bruno  Dept. of Gastroenterology and Hepatology, Erasmus MC  Postbus 2040, 3000 CA, Rotterdam, The Netherlands |
|  | 5c | Role of study sponsor and funders, if any, in study design; collection, management, analysis, and interpretation of data; writing of the report; and the decision to submit the report for publication, including whether they will have ultimate authority over any of these activities | 26 |
|  | 5d | Composition, roles, and responsibilities of the coordinating centre, steering committee, endpoint adjudication committee, data management team, and other individuals or groups overseeing the trial, if applicable (see Item 21a for data monitoring committee) | 26 |
| Introduction |  |  |  |
| Background and rationale | 6a | Description of research question and justification for undertaking the trial, including summary of relevant studies (published and unpublished) examining benefits and harms for each intervention | 5 |
|  | 6b | Explanation for choice of comparators | 5 |
| Objectives | 7 | Specific objectives or hypotheses | 5 |
| Trial design | 8 | Description of trial design including type of trial (eg, parallel group, crossover, factorial, single group), allocation ratio, and framework (eg, superiority, equivalence, noninferiority, exploratory) | 6-7 |
| Methods: Participants, interventions, and outcomes | | |  |
| Study setting | 9 | Description of study settings (eg, community clinic, academic hospital) and list of countries where data will be collected. Reference to where list of study sites can be obtained | 6-7 |
| Eligibility criteria | 10 | Inclusion and exclusion criteria for participants. If applicable, eligibility criteria for study centres and individuals who will perform the interventions (eg, surgeons, psychotherapists) | 8 |
| Interventions | 11a | Interventions for each group with sufficient detail to allow replication, including how and when they will be administered | 9-13 |
|  | 11b | Criteria for discontinuing or modifying allocated interventions for a given trial participant (eg, drug dose change in response to harms, participant request, or improving/worsening disease) | N/A  No criteria are defined for discontinuing or modifying the implementation of our evidence-based management algorithm. There are no additional risks for patients participating in this trial. In this study no experimental drugs or medical devices are used whose effectiveness and safety have not yet been demonstrated. The effectiveness and safety of each of our interventions have already been demonstrated in previous research (i.e. evidence-based). We aim to improve care by implementing these interventions into clinical practice. |
|  | 11c | Strategies to improve adherence to intervention protocols, and any procedures for monitoring adherence (eg, drug tablet return, laboratory tests) | 9-10, 13 |
|  | 11d | Relevant concomitant care and interventions that are permitted or prohibited during the trial | N/A. We do not aim to interfere with concurrent treatments. Furthermore, the treating physician is responsible for the interpretation and implementation of the individualized and standardized treatment plan that follows after using the web-application tool in clinical practice. |
| Outcomes | 12 | Primary, secondary, and other outcomes, including the specific measurement variable (eg, systolic blood pressure), analysis metric (eg, change from baseline, final value, time to event), method of aggregation (eg, median, proportion), and time point for each outcome. Explanation of the clinical relevance of chosen efficacy and harm outcomes is strongly recommended | 14, Supplementary Appendix-4 |
| Participant timeline | 13 | Time schedule of enrolment, interventions (including any run-ins and washouts), assessments, and visits for participants. A schematic diagram is highly recommended (see Figure) | 6-7 (figure 1 and 2) |
| Sample size | 14 | Estimated number of participants needed to achieve study objectives and how it was determined, including clinical and statistical assumptions supporting any sample size calculations | 14-15 |
| Recruitment | 15 | Strategies for achieving adequate participant enrolment to reach target sample size | 20  Additional information not included in the manuscript: Patients who are being treated for chronic pancreatitis in the participating Dutch centers during the inclusion period of this trial will be identified by using the DBC-753 for CP (i.e., each disease has a DBC-specific code which represents a combination of diagnostic and therapeutic interventions needed for disease management). To these patients a letter will be sent by their treating physician to ask for their permission to share their contact details (i.e. name, address and phone number) with the coordinating investigators of this trial. After providing permission, patients will be contacted by the coordinating investigator to provide information about this trial and, if patients are willing to participate, to check for their eligibility. Eligible patients will be asked to complete the informed consent form. Subsequently, eligible CP-patients who are not identified by using the DBC-753-code and patients newly diagnosed with CP will be asked for informed consent by their treating physician during an outpatient clinic visit. After providing written informed consent, the data of diagnosis will be verified with data from patients’ medical records. After 5 months of inclusion, we will perform an interim administrative look to monitor recruitment and retention rate. If less than 75% of the sample size is reached at that time, the duration of time between each step will be prolonged for the remaining clusters so that we will reach our target sample size. |
| **Methods: Assignment of interventions (for controlled trials)** | | |  |
| Allocation: |  |  |  |
| Sequence generation | 16a | Method of generating the allocation sequence (eg, computer-generated random numbers), and list of any factors for stratification. To reduce predictability of a random sequence, details of any planned restriction (eg, blocking) should be provided in a separate document that is unavailable to those who enrol participants or assign interventions | 15 |
| Allocation concealment mechanism | 16b | Mechanism of implementing the allocation sequence (eg, central telephone; sequentially numbered, opaque, sealed envelopes), describing any steps to conceal the sequence until interventions are assigned | 7, 9-10,15 |
| Implementation | 16c | Who will generate the allocation sequence, who will enrol participants, and who will assign participants to interventions | 15 |
| Blinding (masking) | 17a | Who will be blinded after assignment to interventions (eg, trial participants, care providers, outcome assessors, data analysts), and how | 15 |
|  | 17b | If blinded, circumstances under which unblinding is permissible, and procedure for revealing a participant’s allocated intervention during the trial | N/A. There will be no blinding to treatment in this study. Both patients and physicians are aware of all the interventions they are receiving/ providing. |
| **Methods: Data collection, management, and analysis** | | |  |
| Data collection methods | 18a | Plans for assessment and collection of outcome, baseline, and other trial data, including any related processes to promote data quality (eg, duplicate measurements, training of assessors) and a description of study instruments (eg, questionnaires, laboratory tests) along with their reliability and validity, if known. Reference to where data collection forms can be found, if not in the protocol | 16, 26, Supplementary appendix-4 |
|  | 18b | Plans to promote participant retention and complete follow-up, including list of any outcome data to be collected for participants who discontinue or deviate from intervention protocols | 8 |
| Data management | 19 | Plans for data entry, coding, security, and storage, including any related processes to promote data quality (eg, double data entry; range checks for data values). Reference to where details of data management procedures can be found, if not in the protocol | 26 |
| Statistical methods | 20a | Statistical methods for analysing primary and secondary outcomes. Reference to where other details of the statistical analysis plan can be found, if not in the protocol | 16-20 |
|  | 20b | Methods for any additional analyses (eg, subgroup and adjusted analyses) | 17, 19 |
|  | 20c | Definition of analysis population relating to protocol non-adherence (eg, as randomised analysis), and any statistical methods to handle missing data (eg, multiple imputation) | 16-17 |
| **Methods: Monitoring** | | |  |
| Data monitoring | 21a | Composition of data monitoring committee (DMC); summary of its role and reporting structure; statement of whether it is independent from the sponsor and competing interests; and reference to where further details about its charter can be found, if not in the protocol. Alternatively, an explanation of why a DMC is not needed | N/A. This study is no subject to the Medical Research Involving Human Subject act (WMO). Therefore, a data monitoring committee (DMC) is not required according to Dutch law. |
|  | 21b | Description of any interim analyses and stopping guidelines, including who will have access to these interim results and make the final decision to terminate the trial | 20 |
| Harms | 22 | Plans for collecting, assessing, reporting, and managing solicited and spontaneously reported adverse events and other unintended effects of trial interventions or trial conduct | N/A. With the implementation of our management algorithm we aim to improve adherence to the HaPanEU-guidelines. All interventions that need to be implemented have been studied in previous studies and proved no harm. Furthermore, these interventions should already be part of standard practice according to these current guidelines. Therefore, no registry is set up for this trial to report and register (serious) adverse events. If complications after surgery/endoscopic treatment do occur, these events should be registered and reported by the treating physician/hospital as a complication of their regular treatment. |
| Auditing | 23 | Frequency and procedures for auditing trial conduct, if any, and whether the process will be independent from investigators and the sponsor | In this trial there will be no audit procedures performed. |
| Ethics and dissemination | | |  |
| Research ethics approval | 24 | Plans for seeking research ethics committee/institutional review board (REC/IRB) approval | 27 |
| Protocol amendments | 25 | Plans for communicating important protocol modifications (eg, changes to eligibility criteria, outcomes, analyses) to relevant parties (eg, investigators, REC/IRBs, trial participants, trial registries, journals, regulators) | All amendments will be notified to the METC that gave a favorable opinion. |
| Consent or assent | 26a | Who will obtain informed consent or assent from potential trial participants or authorised surrogates, and how (see Item 32) | 27 |
|  | 26b | Additional consent provisions for collection and use of participant data and biological specimens in ancillary studies, if applicable | 27 |
| Confidentiality | 27 | How personal information about potential and enrolled participants will be collected, shared, and maintained in order to protect confidentiality before, during, and after the trial | 26 |
| Declaration of interests | 28 | Financial and other competing interests for principal investigators for the overall trial and each study site | 26 |
| Access to data | 29 | Statement of who will have access to the final trial dataset, and disclosure of contractual agreements that limit such access for investigators | 26 |
| Ancillary and post-trial care | 30 | Provisions, if any, for ancillary and post-trial care, and for compensation to those who suffer harm from trial participation | There are no additional risks for patients participating in this trial. Subjects will not be offered any incentives to participate in this study. |
| Dissemination policy | 31a | Plans for investigators and sponsor to communicate trial results to participants, healthcare professionals, the public, and other relevant groups (eg, via publication, reporting in results databases, or other data sharing arrangements), including any publication restrictions | 26 |
|  | 31b | Authorship eligibility guidelines and any intended use of professional writers | 26 |
|  | 31c | Plans, if any, for granting public access to the full protocol, participant-level dataset, and statistical code | 26 |
| Appendices |  |  |  |
| Informed consent materials | 32 | Model consent form and other related documentation given to participants and authorised surrogates | On request |
| Biological specimens | 33 | Plans for collection, laboratory evaluation, and storage of biological specimens for genetic or molecular analysis in the current trial and for future use in ancillary studies, if applicable | Not applicable. No biological specimens or genetic material will be obtained from participants during this trial. |

*It is strongly recommended that this checklist be read in conjunction with the SPIRIT 2013 Explanation & Elaboration for important clarification on the items. Amendments to the protocol should be tracked and dated. The SPIRIT checklist is copyrighted by the SPIRIT Group under the Creative Commons “[Attribution-NonCommercial-NoDerivs 3.0 Unported](http://www.creativecommons.org/licenses/by-nc-nd/3.0/)” license.

# SUPPLEMENTARY APPENDIX 2: SYSTEMATIC LITERATURE SEARCH

## Life style modifications

**Search**

A systematic search in PubMed and EMBASE databases was performed for studies reporting on alcohol and smoking behavior of chronic pancreatitis patients. The search was performed on December 3th, 2019 with the following items in both Mesh terms and plain text; 1; (alcoholism) OR (ethanol) OR (alcohol abstinence) OR (alcohol) OR (alcohol drinking) OR (alcohol drinking habit) OR (nicotine) OR (tobacco) OR (smoking) OR (smoking cessation) AND 2; (chronic pancreatitis).

**Search PubMed:**

("Pancreatitis, Chronic"[Mesh] OR "chronic pancreatitis"[Title/Abstract]) AND ("Alcoholism"[Mesh] OR "Ethanol"[Mesh] OR "Alcohol Abstinence"[Mesh] OR alcohol[Title/Abstract] OR "Alcohol Drinking"[Mesh] OR alcohol drinking habit*[Title/Abstract] OR alcohol abstinence [Title/Abstract] OR ethanol[Title/Abstract]) OR ("Nicotine"[Mesh] OR smoking cessation[Title/Abstract] OR "Tobacco"[Mesh] OR "Smoking"[Mesh] OR nicotine[Title/Abstract] OR tobacco[Title/Abstract] OR smoking[Title/Abstract] OR "Smoking Cessation"[Mesh]) NOT (animals[MeSH] NOT humans[MeSH])

**Result: 1.985 articles**

Articles published between January 1^st^ of 2016 and 3th of December 2019: **282 articles

Search EMBASE:**

('chronic pancreatitis'/exp OR 'chronic pancreatitis':ab,ti) AND (('smoking cessation'/exp OR 'smoking'/exp OR 'nicotine'/exp OR 'tobacco use'/exp OR 'tobacco'/exp OR smoking:ab,ti OR nicotine:ab,ti OR tobacco:ab,ti) OR 'alcohol'/exp OR 'alcoholism'/exp OR 'alcohol abstinence'/exp OR 'alcohol consumption'/exp OR alcohol:ab,ti OR ethanol:ab,ti) NOT (('animal experiment'/exp OR 'animal model'/exp OR 'nonhuman'/exp) NOT 'human'/exp)

**Result: 4.439 articles**

Articles published between January 1^st^ of 2016 and 3th of December 2019: **1.047 articles**

**In total: 1.329 articles**After removing duplicates: 1.023 articles left

Title- and abstract screening: 17 articles left 🡪 whereof 9 articles were not available or conference abstracts 🡪 8 articles were eligible for full-text screening

**Predefined in- and exclusion criteria:**

Inclusion criteria: 1) including chronic pancreatitis patients; 2) reporting on alcohol and/or smoking behaviour in chronic pancreatitis patients

Exclusion criteria: 1) animal studies; 2) studies reporting on a specific subgroup of CP-patients; 3) studies reporting in other than English language; 4) unpublished studies and conference abstracts; 5) review studies reporting on the overall management of chronic pancreatitis

## Exocrine pancreatic insufficiency

**Search**
A systematic search in PubMed and EMBASE databases was performed for studies reporting on exocrine pancreatic insufficiency in chronic pancreatitis patients. The search was performed on November 14, 2019 with the following items in both Mesh terms and plain text; 1; (exocrine pancreatic insufficiency) OR (exocrine) OR (pancreatic funct*) OR (pancreas funct*) OR (pancreatic dysfunct*) OR (pancreas dysfunct*) OR (pancreatic insufficien*) OR (pancreas insufficien*) AND 2; (chronic pancreatitis).

**Search PubMed:**

("Exocrine pancreatic insufficiency"[Mesh] OR exocrine[tiab] OR pancreatic funct*[tiab] OR pancreas funct*[tiab] OR pancreatic dysfunct*[tiab] OR pancreas dysfunct*[tiab] OR pancreatic insufficien*[tiab] OR pancreas insufficien*[tiab]) AND ("Pancreatitis, Chronic"[Mesh] OR "chronic pancreatitis"[tiab]) NOT (animals[mesh] NOT humans[Mesh])

**Result: 2.062 articles**

Articles published between January 1^st^ of 2016 and 14^th^ of November 2019: **296 articles

Search EMBASE:**

'pancreas exocrine insufficiency'/exp OR 'pancreas function'/exp OR exocrine:ab,ti OR ((pancreatic OR pancreas) NEXT/1 (funct* OR dysfunct* OR insufficien*)):ab,ti AND ('chronic pancreatitis'/exp OR 'chronic pancreatitis':ab,ti) NOT ('animal experiment'/exp OR 'animal model'/exp OR 'nonhuman'/exp NOT 'human'/exp)

**Result: 3.851 articles**

Articles published between January 1^st^ of 2016 and 14^th^ of November 2019: **744 articles**

**In total: 1.040 articles**

After removing duplicates: 751 articles left

Title- and abstract screening: 40 articles left 🡪 whereof 17 articles were not available or conference abstracts 🡪 23 articles were eligible for full-text screening

**Predefined in- and exclusion criteria:**

Inclusion criteria: 1) including chronic pancreatitis patients; 2) reporting on exocrine pancreatic insufficiency in chronic pancreatitis patients

Exclusion criteria: 1) animal studies; 2) studies reporting on a specific subgroup of CP-patients; 3) studies reporting on exocrine pancreatic insufficiency after surgery in chronic pancreatitis patients/patients with pancreas carcinoma; 4) studies reporting in other than English language; 5) unpublished studies and conference abstracts; 6) review studies reporting on the overall management of chronic pancreatitis

## Endocrine pancreatic insufficiency

**Search**

A systematic search in PubMed and EMBASE databases was performed for studies reporting on diabetes mellitus type 3c in chronic pancreatitis patients. The search was performed on December 29, 2019 with the following items in both Mesh terms and plain text; 1; (diabetes mellitus*) OR (type 3c diabetes) OR (diabetes type 3c) OR (type IIIc diabetes) OR (diabetes type IIIc) OR (pancreatogenic diabetes) OR (diabetes) OR (T3cDM) OR (endocrine pancreatic insufficiency) OR (endocrine dysfunct*) AND 2; (chronic pancreatitis).

**Search PubMed:**

("Diabetes Mellitus"[Mesh] OR diabetes mellitus*[tiab] OR type 3c diabetes[tiab] OR diabetes type 3c [tiab] OR type IIIc diabetes [tiab] OR diabetes type IIIc [tiab] OR "pancreatogenic diabetes"[tiab] OR diabetes [tiab] OR T3cDM [tiab] OR "endocrine pancreatic insufficiency"[tiab] OR endocrine dysfunct*[tiab]) AND ("Pancreatitis, Chronic"[Mesh] OR "chronic pancreatitis"[tiab]) NOT (animals[MeSH] NOT humans[MeSH])

**Result: 1.660 articles**

Articles published between January 1^st^ of 2016 and 29^th^ of December 2019: **328 articles**

**Search EMBASE:**

(('diabetes mellitus'/exp OR 'diabetes mellitus':ab,ti OR 'type 3c diabetes':ab,ti OR 'diabetes type 3c':ab,ti OR 'type iiic diabetes':ab,ti OR 'diabetes type iiic':ab,ti OR 'pancreatogenic diabetes':ab,ti OR 'diabetes':ab,ti OR 't3cdm':ab,ti OR 'endocrine pancreatic insufficiency':ab,ti OR 'endocrine dysfunct*':ab,ti) AND ('chronic pancreatitis'/exp OR 'chronic pancreatitis':ab,ti)) NOT (('animal experiment'/exp OR 'animal model'/exp OR 'nonhuman'/exp) NOT 'human'/exp)

**Result: 3.567 articles**

Articles published between January 1^st^ of 2016 and 29^th^ of December 2019: **959 articles**

**In total: 1.287 articles**After removing duplicates: 1.228 left

Title- and abstract screening: 21 articles left 🡪 whereof 12 articles not available or conference abstracts 🡪 9 articles were eligible for full-text screening

**Predefined in- and exclusion criteria:**

Inclusion criteria: 1) including chronic pancreatitis patients; 2) reporting on endocrine pancreatic insufficiency in chronic pancreatitis patients

Exclusion criteria: 1) animal studies; 2) studies reporting on a specific subgroup of CP-patients; 3) studies reporting on endocrine pancreatic insufficiency before/after surgery in chronic pancreatitis patients/patients with pancreas carcinoma; 4) studies reporting in other than English language; 5) unpublished studies and conference abstracts; 6) review studies reporting on the overall management of chronic pancreatitis

## Nutritional status

**Search**

A systematic search in PubMed and EMBASE databases was performed for studies reporting on nutritional management in chronic pancreatitis patients. The search was performed on January 8th, 2020 with the following items in both Mesh terms and plain text;

1; (nutritional status) OR (nutrition therapy) OR (nutrition assessment) OR (enteral nutrition) OR (parenteral nutrition) OR (nutrition*) OR (malnutrition) AND 2; (chronic pancreatitis).

**Search PubMed:**

("Nutritional Status"[Mesh] OR “Nutrition Therapy”[Mesh] OR “Nutrition Assessment”[Mesh] OR “enteral nutrition”[tiab] OR “parenteral nutrition”[tiab] OR “malnutrition”[Mesh] OR “nutritional status”[tiab] OR “nutrition therapy”[tiab] OR “nutrition assessment”[tiab] OR nutrition* [tiab] OR malnutrition[tiab]) AND ("Pancreatitis, Chronic"[Mesh] OR "chronic pancreatitis"[tiab]) NOT (animals[MeSH] NOT humans[MeSH])

**Result: 609 articles**

Articles published between January 1^st^ of 2016 and 8^th^ of January 2020: **102 articles**

**Search EMBASE:**

('nutritional status'/exp OR 'diet therapy'/exp OR 'nutrition'/exp OR 'nutritional assessment'/exp OR 'malnutrition'/exp OR ‘artificial feeding’/exp OR 'nutritional status':ab,ti OR 'diet therapy':ab,ti OR 'nutritional therapy':ab,ti OR 'nutrition assessment':ab,ti OR 'malnutrition':ab,ti OR 'nutrition':ab,ti) AND ('chronic pancreatitis'/exp OR 'chronic pancreatitis':ab,ti) NOT (('animal experiment'/exp OR 'animal model'/exp OR 'nonhuman'/exp) NOT 'human'/exp)

**Result: 3.977 articles**

Articles published between January 1^st^ of 2016 and 8^th^ of January 2020: **977 articles**

**In total: 1.079 articles**After removing duplicates: 940 left

Title- and abstract screening: 20 articles left 🡪 whereof 9 articles were not available or conference abstracts or a thesis, 1 article was found through cross-linking 🡪 11 articles were eligible for full-text screening

**Predefined in- and exclusion criteria:**

Inclusion criteria: 1) including chronic pancreatitis patients; 2) reporting on nutritional management in chronic pancreatitis patients

Exclusion criteria: 1) animal studies; 2) studies reporting on a specific subgroup of CP-patients; 3) studies reporting on nutritional management before/after surgery in chronic pancreatitis patients/patients with pancreas carcinoma; 4) studies reporting in other than English language; 5) unpublished studies, theses and conference abstracts; 6) review studies reporting on the overall management of chronic pancreatitis

Reference lists of all included publications were screened for relevant studies reporting on nutritional management in chronic pancreatitis patients that were not identified by our primary search strategy (cross-linking).

## Bone health

**Search**

A systematic search in PubMed and EMBASE databases was performed for studies reporting on bone health of chronic pancreatitis patients. The search was performed on January 8th, 2020 with the following items in both Mesh terms and plain text;

1; (bone diseases) OR (bone remodeling) OR (bone density) OR (osteoporosis) OR (osteopenia) OR (osteopaenia) OR (bone health) OR (bone disease*) AND 2; (chronic pancreatitis).

**Search PubMed:**

("Bone Diseases"[Mesh]) OR “Bone Remodeling”[Mesh] OR “osteoporosis”[Mesh] OR “bone density”[Mesh] OR osteoporosis[tiab] OR bone disease*[tiab] OR osteopenia[tiab] OR osteopaenia[tiab] OR bone health [tiab] OR “bone remodeling”[tiab] OR "bone density"[tiab]) AND ("Pancreatitis, Chronic"[Mesh] OR "chronic pancreatitis"[tiab]) NOT (animals[MeSH] NOT humans[MeSH])

**Result: 97 articles**

Articles published between January 1^st^ of 2016 and 8^th^ of January 2020: **25 articles**

**Search EMBASE:**

('bone health'/exp OR 'bone density'/exp OR 'bone disease'/exp OR 'bone remodeling'/exp OR 'osteoporosis'/exp OR 'osteopenia'/exp OR 'bone health':ab,ti OR 'bone disease*':ab,ti OR 'bone remodeling':ab,ti OR 'osteoporosis':ab,ti OR 'osteopenia':ab,ti OR 'osteopaenia':ab,ti OR 'bone density':ab,ti) AND ('chronic pancreatitis'/exp OR 'chronic pancreatitis':ab,ti) NOT (('animal experiment'/exp OR 'animal model'/exp OR 'nonhuman'/exp) NOT 'human'/exp)

**Result: 373 articles**

Articles published between January 1^st^ of 2016 and 8^th^ of January 2020: **130 articles**

**In total: 155 articles**After removing duplicates: 127 left

Title- and abstract screening: 19 articles left 🡪 whereof 14 articles were not available or conference abstracts 🡪 5 articles were eligible for full-text screening

**Predefined in- and exclusion criteria:**

Inclusion criteria: 1) including chronic pancreatitis patients; 2) reporting on bone health of chronic pancreatitis patients

Exclusion criteria: 1) animal studies; 2) studies reporting on a specific subgroup of CP-patients; 3) studies reporting in other than English language; 4) unpublished studies and conference abstracts; 5) review studies reporting on the overall management of chronic pancreatitis

## Pain management

**Search**

A systematic search in PubMed and EMBASE databases was performed for studies reporting on pain in chronic pancreatitis patients. The search was performed on November 22, 2019 with the following items in both Mesh terms and plain text; 1; (pain) OR (chronic pain) OR (pain management) OR (abdominal pain) AND 2; (chronic pancreatitis).

**Search PubMed:**

(“Pain”[Mesh] OR pain [tiab] OR “Chronic Pain”[Mesh] OR “Chronic Pain*”[tiab] OR “Pain Management”[Mesh] OR “Pain Management”[tiab] OR “abdominal pain”[Mesh] OR “abdominal pain*”[tiab] AND (“Pancreatitis, Chronic”[Mesh] OR “chronic pancreatitis”[tiab]) NOT (animals[Mesh] NOT humans [Mesh])

**Result: 2.897 articles**

Articles published between January 1^st^ of 2016 and 22^th^ of November 2019: **446 articles

Search EMBASE:**

('chronic pancreatitis'/exp OR 'chronic pancreatitis':ab,ti) AND ('pain'/exp OR 'chronic pain'/exp OR 'pain therapy'/exp OR ‘pain therapies’:ab,ti OR 'pain':ab,ti OR 'chronic pain':ab,ti OR 'pain management':ab,ti OR 'abdominal pain':ab,ti OR 'pain therapy':ab,ti) NOT (('animal experiment'/exp OR 'animal model'/exp OR 'nonhuman'/exp) NOT 'human'/exp)

**Result: 6.301 articles**

Articles published between January 1^st^ of 2016 and 14^th^ of November 2019: **1.484 articles**

**In total: 1.930 articles**After removing duplicates: 1.494 left

Title- and abstract screening: 27 articles left 🡪 whereof 6 articles were not available or conference abstracts 🡪 21 articles were eligible for full-text screening

**Predefined in- and exclusion criteria:**

Inclusion criteria: 1) including chronic pancreatitis patients; 2) reporting on pain in chronic pancreatitis patients

Exclusion criteria: 1) animal studies; 2) studies reporting on a specific subgroup of CP-patients; 3) studies reporting on chronic pancreatitis patients with pancreas carcinoma or surgical strategies in patients with pancreas carcinoma; 4) studies reporting in other than English language; 5) unpublished studies, theses and conference abstracts; 6) review studies reporting on the overall management of chronic pancreatitis

During the writing process of this study protocol a new study reporting on new international consensus guidelines for surgery and the timing of intervention in chronic pancreatitis was published. The findings of this study have been included in the management algorithm. The reference list of this article was screened for relevant studies reporting on pain management in chronic pancreatitis patients that were not identified by our primary search strategy (cross-linking).

# SUPPLEMENTARY APPENDIX 3: EVIDENCE-BASED ALGORITHM

## Exocrine pancreatic function


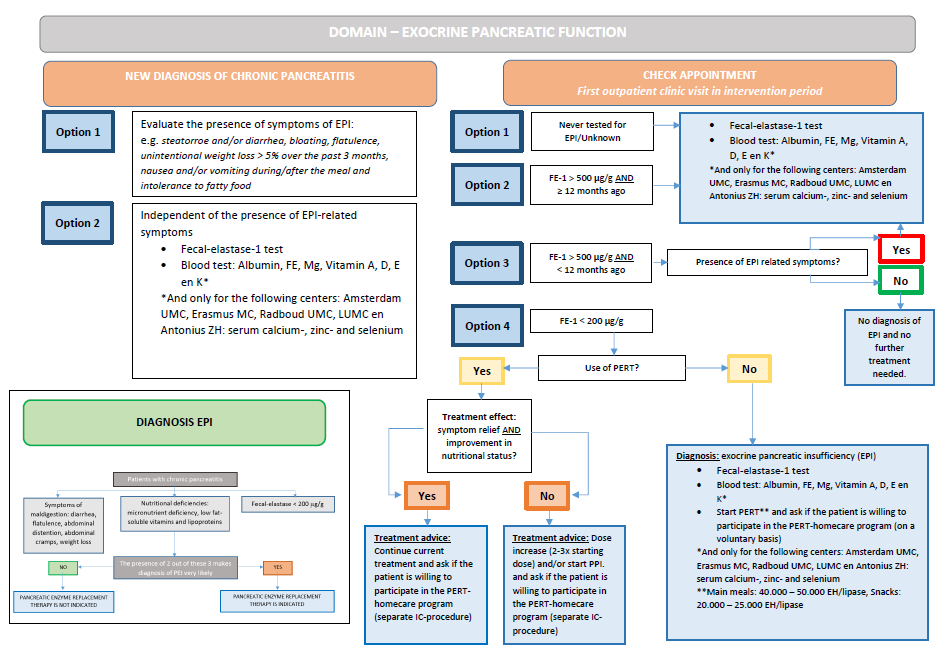


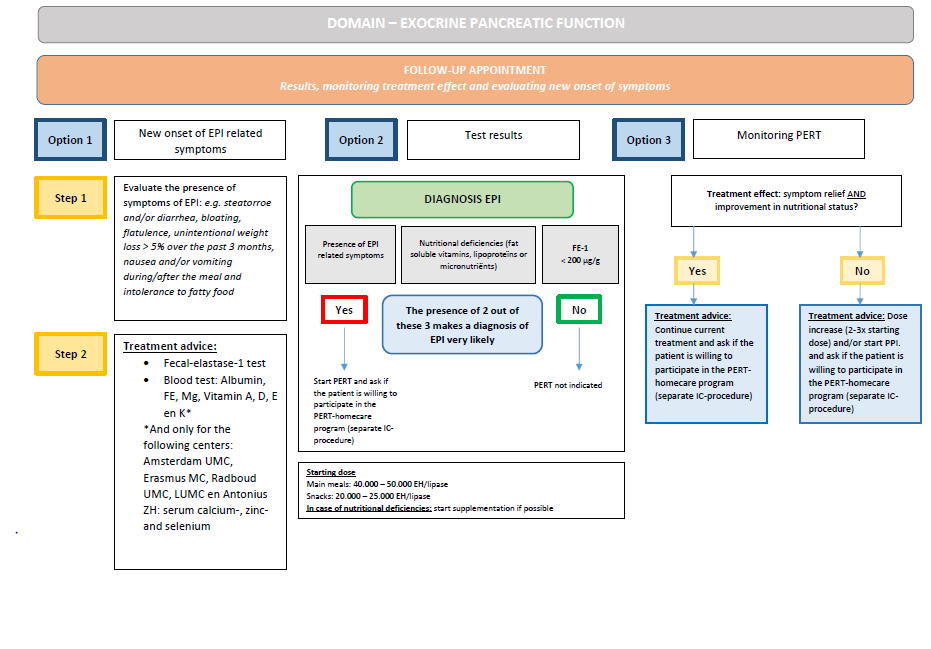


## Endocrine pancreatic function


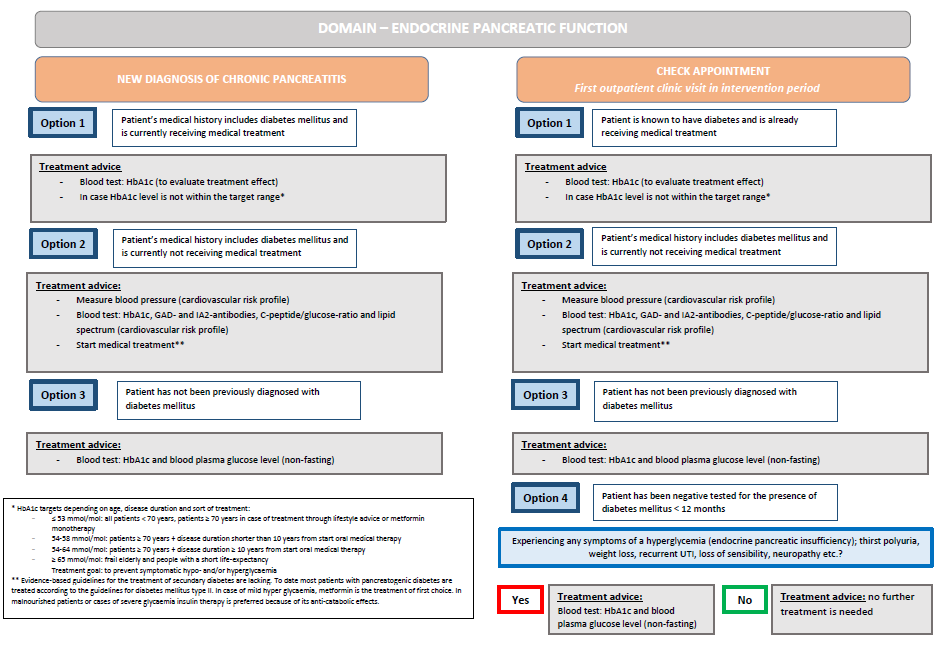


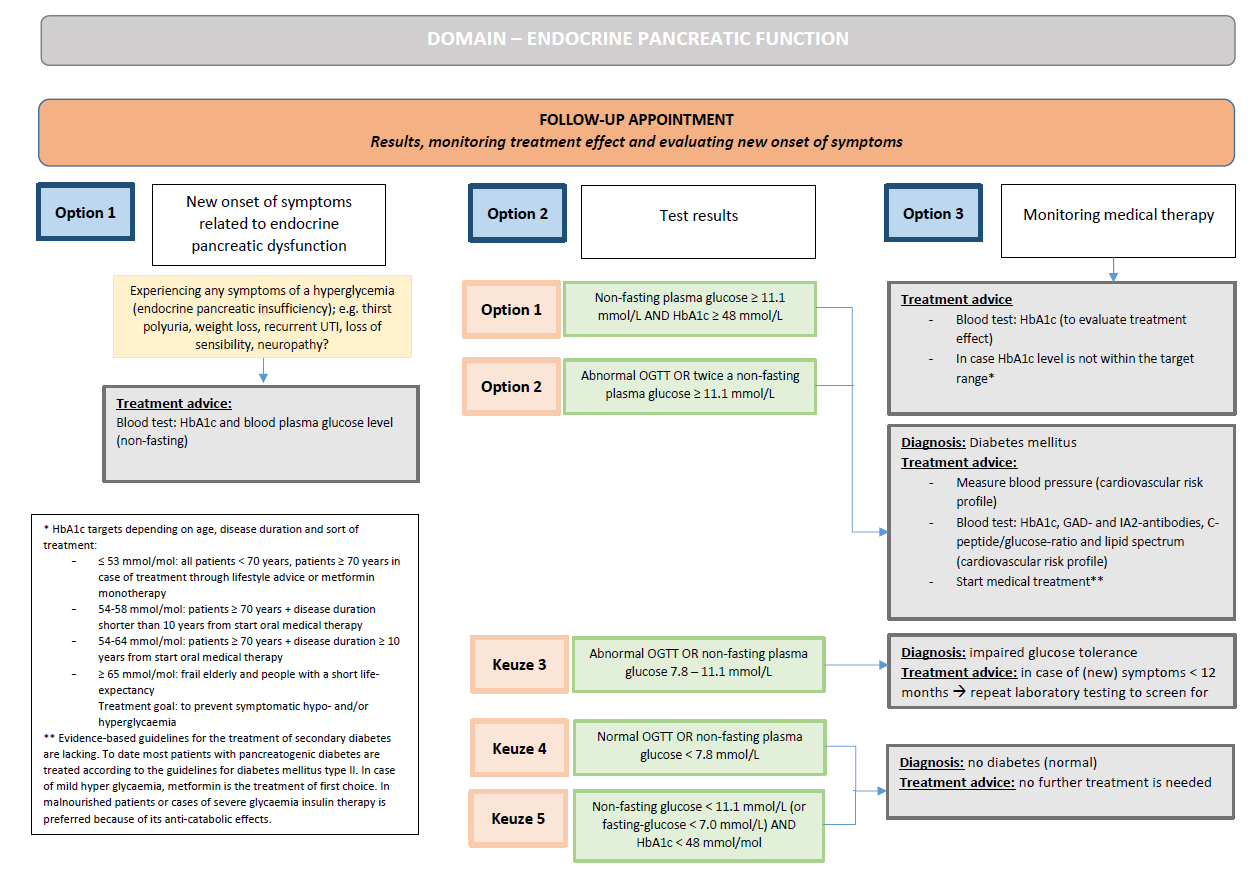


##
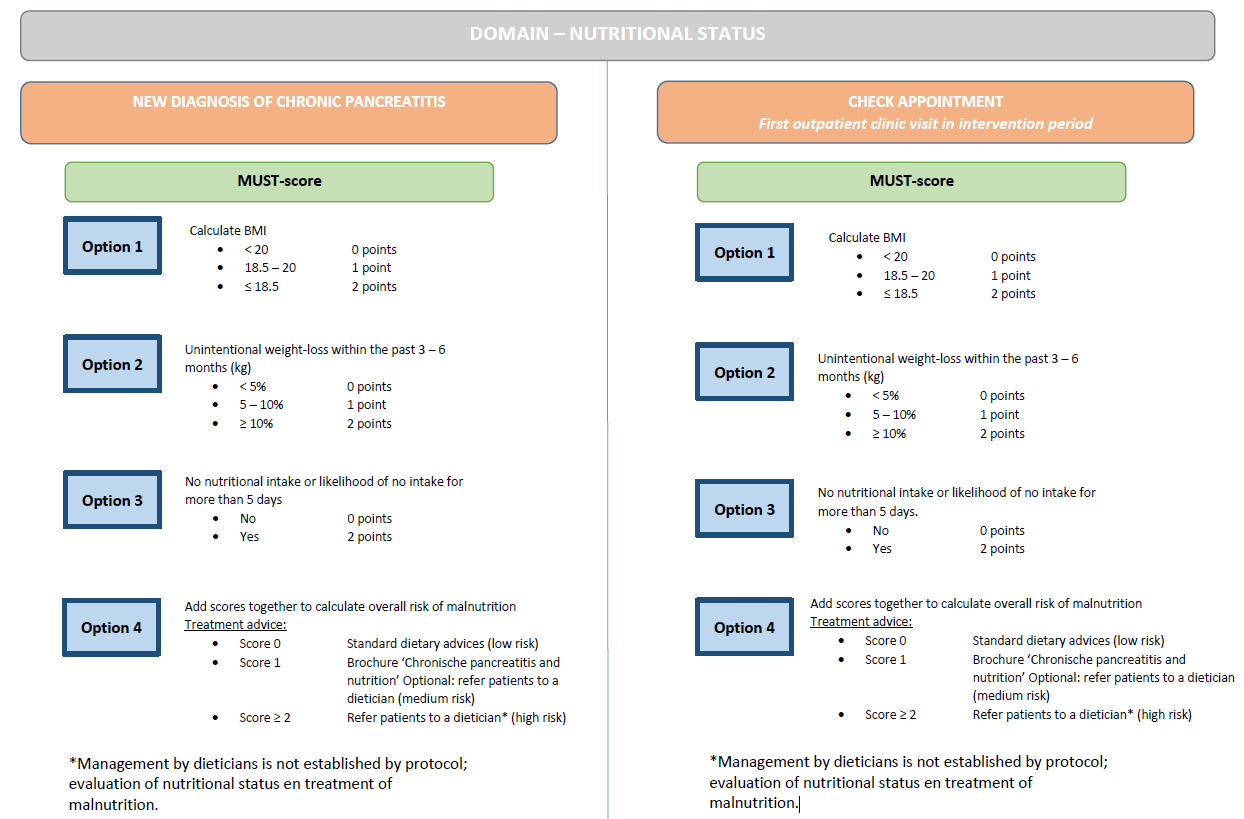
Nutritional status


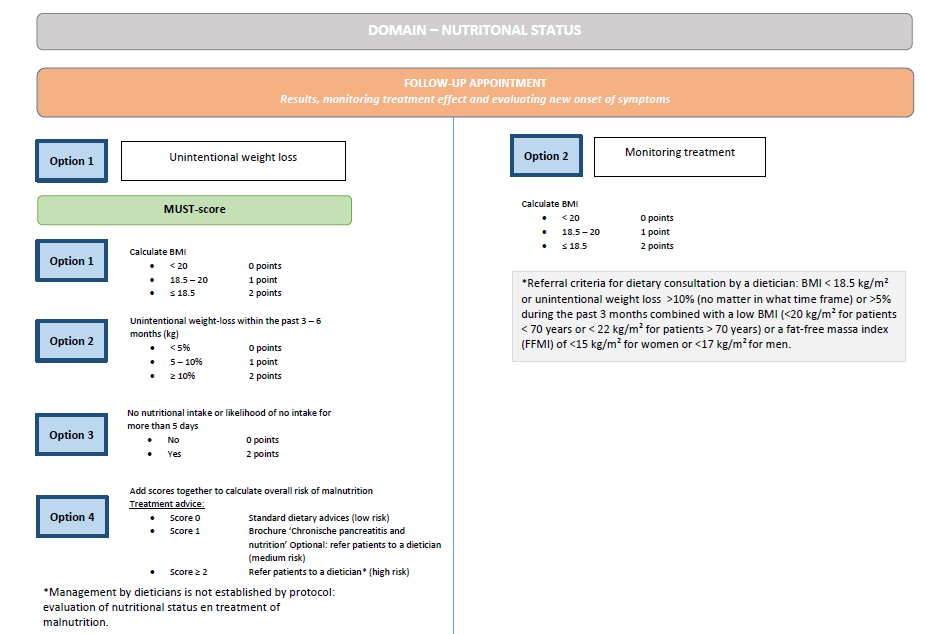


## Bone health


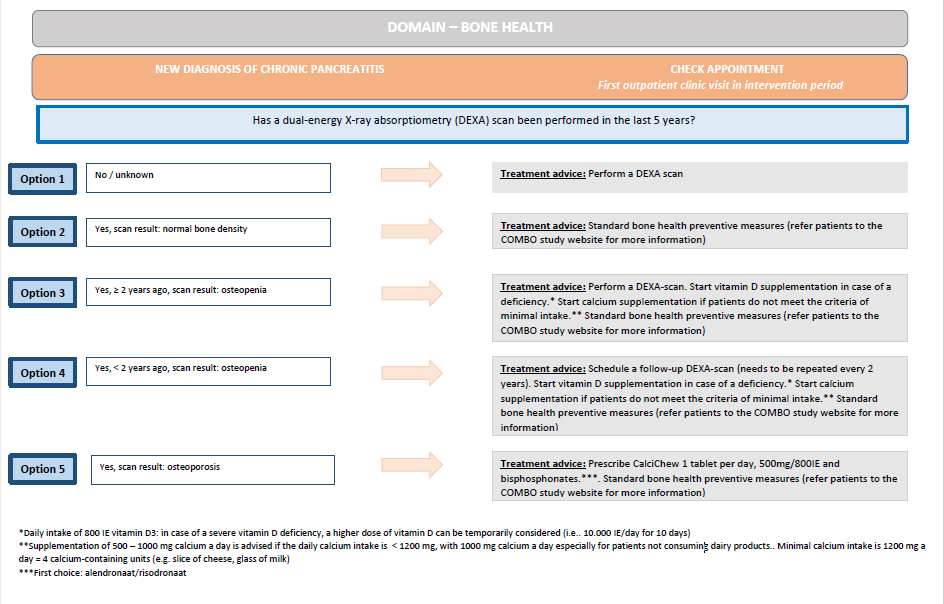


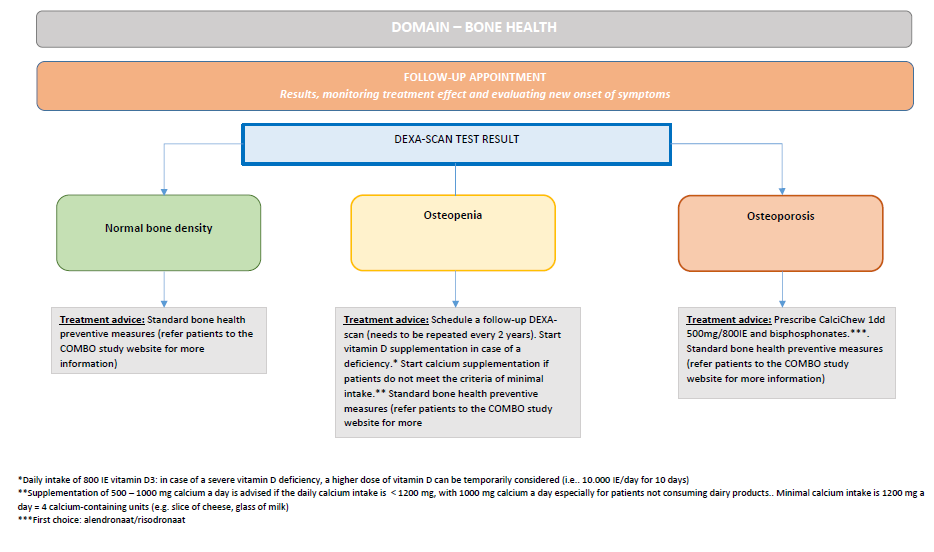


## Pain management


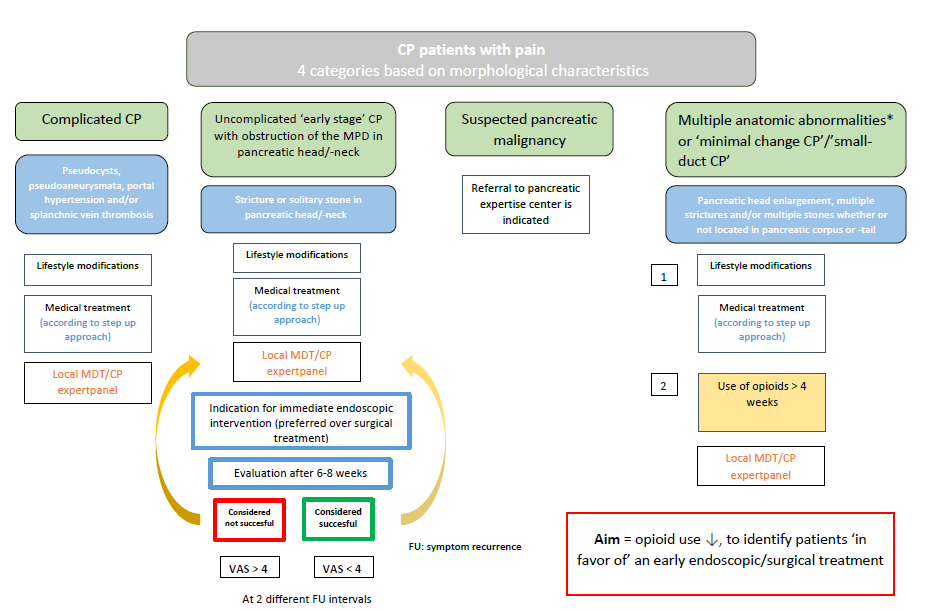


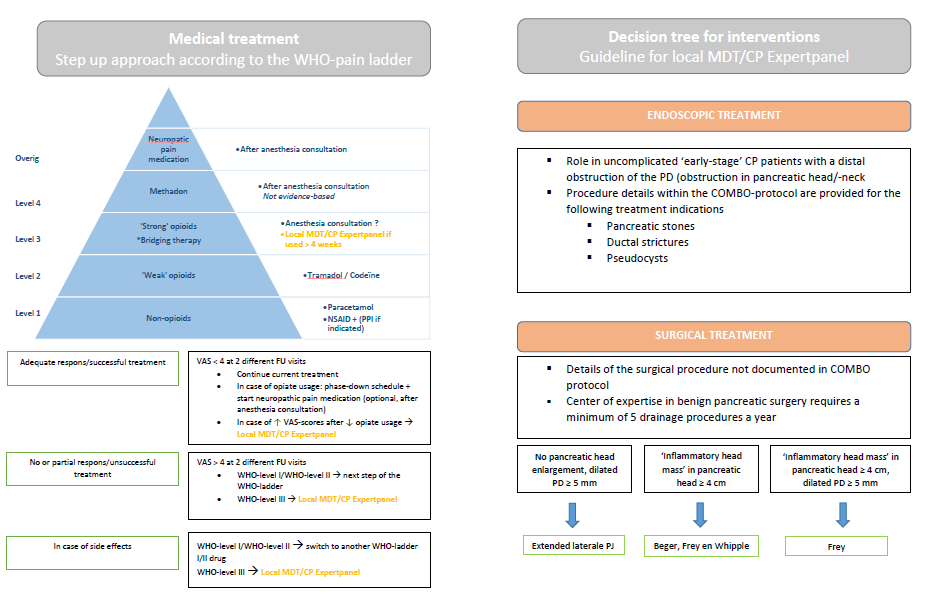


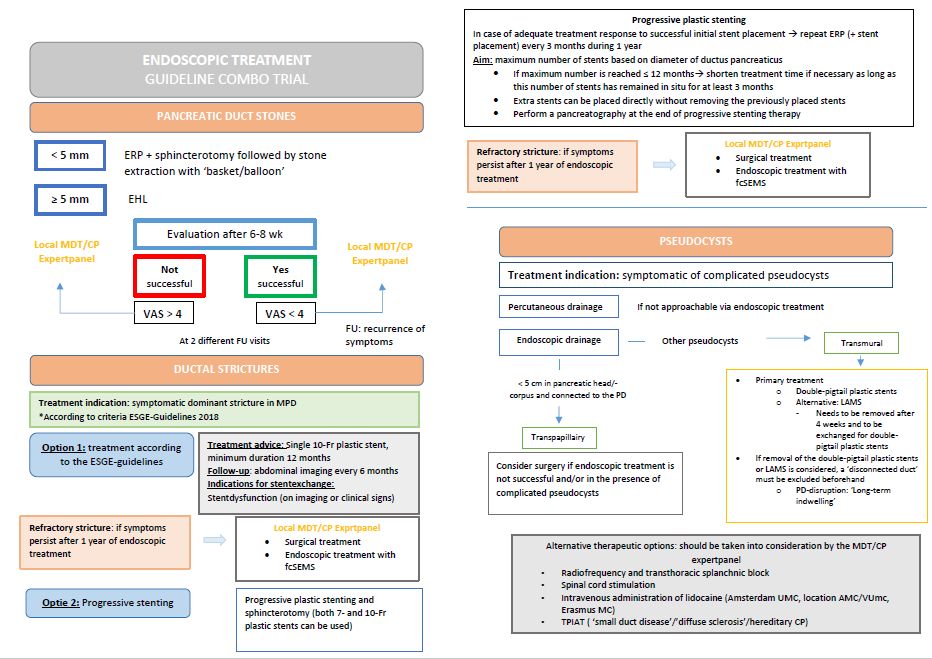


# SUPPLEMENTARY APPENDIX 4: SECONDARY ENDPOINTS

Secondary outcomes will be collected through comprehensive questionnaires or (retrospectively) obtained from the medical records of the included patients at 12 months after the end of the transition period for each cluster (intention-to-treat analysis) and at 12 months after start intervention for the individual patient defined as the first outpatient clinic visit after the kick-off meeting has been performed (per-protocol analysis). Secondary endpoints include individual components of the co-primary endpoints, process measure outcomes, other clinical outcomes, utilization of healthcare resources, social participation and total amount of direct and indirect costs (for details see chapter 8 ‘Statistical analysis’).

**Process measure outcomes**

Process measure outcomes will be obtained to determine adherence to the algorithm (success of implementation). Variables are dichotomous and descriptive analyses will be applied.

- Referral of patients eligible to participate in a smoking- or alcohol cessation program (Smoking: Zorgprofiel 2 and -3, Alcohol: Zorgprofiel 2, -3 and -4)
- Screening for exocrine pancreatic insufficiency (annually or in case of symptoms)
- Screening for deficiencies of fat-soluble vitamins (annually or in case of clinical signs of malnutrition/exocrine pancreatic insufficiency)
- Screening for deficiencies of minerals and plasma proteins (annually or in case of clinical signs of malnutrition/exocrine pancreatic insufficiency)
- Supplementation of exogenous pancreatic enzymes in patients with exocrine pancreatic insufficiency
- Participation of pancreatic enzyme replacement therapy (PERT)-users in the PERT-homecare program of this trial
- Screening for endocrine pancreatic insufficiency/diabetes mellitus type 3c (annually or in case of symptoms)
- Dietician consultation in eligible patients (MUST-score ≥ 2)
- Screening of eligible patients for bone health diseases by performing a dual-energy X-ray absorptiometry (DXA)
- Vitamin D supplementation in eligible patients
- Medical treatment of osteoporosis
- Consultation of the CP Expert-Panel for eligible patients (complicated CP, patients who require opioid therapy, failed endoscopic intervention, certain anatomical abnormalities and VAS ≥ 4 after an endoscopic/surgical intervention)

**Other clinical outcomes**

- Other quality of life-scores: SF-36, EQ5D
- Lifestyle
  - Alcohol Use Disorders Identification Test (AUDIT)
  - Karl-Fagerstrom Test for Nicotine Dependence (FNTD)
- Pancreatic function
  - Exocrine pancreatic insufficiency: fecal elastase levels, nutritional deficiencies and symptoms of maldigestion (presence of gastro-intestinal symptoms related to exocrine pancreatic insufficiency)
  - Endocrine pancreatic insufficiency: determined by an abnormal serum glucose level (fasting plasma glucose > 7.0 mmol/L or random plasma glucose > 11.1 mmol/L) confirmed by repeat testing, an abnormal HbA1c > 6.5% or an abnormal oral glucose tolerance test (OGTT)
  - Adverse effects of pancreatic enzyme replacement therapy
  - PERT: average daily dose
  - Oral antidiabetic medication: quantity, types and dosages
  - Insulin therapy: average daily dose
- Nutritional status and support
  - Deficiencies of fat-soluble vitamins (A, D, E and K)
  - Deficiencies of minerals and plasma proteins (magnesium, iron, calcium, zinc, selenium and albumin)
  - Malnutrition Universal Screening Tool (MUST)
  - Body-mass Index (BMI)
  - Supplementation of fat-soluble vitamins (A, D, E and K)
  - Oral nutrition supplements
  - Enteral nutrition supplementation
  - Parenteral nutrition supplementation
- Bone health
  - Osteopenia (T-score < 2.5 SD and -1.0 SD)
  - Osteoporosis (T-score ≤ -2.5)
  - CaD3-supplementation
  - Antiresorptive agents: types and dosages
- Pain management
  - Analgesics: types and dosages
  - Endoscopic interventions, including date and specification
  - Surgical interventions, including date and specification
  - Alternative interventional strategies: including date and specification
- Other medical agents
- Social participation
  - Days of sick leave

**Healthcare research utilization**

- Number and duration of hospital admissions
- Total length of hospital stay in days
- Number of outpatient clinic visits
- Number of pancreatitis flare-up during study period: upper abdominal pain requiring hospitalization with either an increased amylase (> 3 times the upper limit of normal) or typical upper abdominal pain recognized by patient from previous episodes
- Number of performed endoscopic interventions
- Number of performed surgical interventions

**Other study parameters**

- Center specific data
  - Hospital setting
  - Hospital size
  - Number of CP threating physicians
  - Size of the CP-population
  - Annual number of endoscopic interventions for CP
  - Annual number of surgical interventions for CP
- Demographic and clinical patient characteristics
  - Age, years
  - Sex
  - Length, cm
  - Weight, kg
  - Education level
  - Employment/Unemployment
  - Etiology of CP
  - Diagnosis date
  - Medical history

# SUPPLEMENTARY APPENDIX 5: SAMPLE SIZE CALCULATION

We used data from the Dutch Chronic Pancreatitis Registry (CARE) to determine the intra-subject variability and random error for the Izbicki score by fitting the following mixed effects model:

Izbicki ~ daysSinceDiagnosis + (1 + daysSinceDiagnosis | PatientNr)

This model had an acceptable model fit. See below for the parameters from this model used in the sample size simulations. We ran simulations with 2000 iterations to determine the power of the trial design. Design choices for the simulation were:

- Patients had a time since diagnosis ≤ 3 years at the time of the start of intervention phase
- 6 cluster groups each consisting of 4 hospitals
- 6 months of patient enrollment in the first cluster
- 2 months between each cross-over
- 18 months of post-intervention measurement
- A questionnaire is completed every 3 months

Based on CARE data the following assumptions were made:

- Mean pain score at diagnosis (intercept): 37.9
- Mean time-slope of -2.5/365 days
- Random effects:
  - Between-patient variability (SD) for intercept: 26.3
  - Between-patient variability (SD) for time-slope: 3.9/365 days
  - Correlation of random intercept and slope: -0.4
  - Residual error: 13.0
- The following additional assumptions were made:
  - Random effects:
    - Between-cluster variability (SD) for intercept: 5
    - No auto-correlation was assumed
  - Intervention effect:
    - Immediate effect: 0
    - Slope-change: variable and differs from subject to subject
    - Average slope-change: 10 points reduction within 365 days
    - Between-patient variability (SD) for slope-change: 5/365 days
    - Correlation of random intercept and random-slope change: -0.5
- 20% of observations is randomly missing

Based on this design, with 120 subjects the power to demonstrate an average slope change of 10 points reduction after 365 days is > 90 %.
